# Supplementary material for: Creating new layered structures at high pressures: SiS2
Source: Sci Rep. 2016 Nov 25;6:37694. doi: 10.1038/srep37694 (PMC5123579; doi:10.1038/srep37694)
Supplement: Supplementary Information [file srep37694-s1.pdf]

Supplementary Information

Creating new layered structures at high pressures: SiS_2

Dušan Plašienka[1,*], Roman Martoňák[1] and Erio Tosatti[2,3]

1 Department of Experimental Physics, Comenius University, Mlynská Dolina F2, 842 48 Bratislava, Slovakia

2 International School for Advanced Studies (SISSA) and CNR-IOM Democritos, Via Bonomea 265, 34136 Trieste, Italy

3 The Abdus Salam International Centre for Theoretical Physics (ICTP), Strada Costira 11, 34151 Trieste, Italy

* plasienka@fmph.uniba.sk

Structural parameters of layered octahedral phases

phase symmetry (prototype)	unit-cell parameters [Å]	Z	Wyckoff positions	density [g.cm^{-3}]	PBE band gap [eV]
$P\bar{3}m1$ (#164) trigonal (CdI_2-type)	$a = 3.213$<br>$c = 5.310$	1	Si1 1b 0 0 1/2<br>S1 2d 1/3 2/3 0.2521	3.226	0.9
$P6_3mc$ (#186) hexagonal	$a = 3.213$<br>$c = 10.629$	2	Si1 2b 1/3 2/3 0.8799<br>S1 2b 2/3 1/3 0.0035<br>S3 2a 0 0 0.2559	3.223	0.7
$R\bar{3}m$ (#166) trigonal ($CdCl_2$-type)	$a = 3.213$<br>$c = 16.052$	3	Si1 3a 2/3 1/3 1/3<br>S1 6c 1/3 2/3 0.4151	3.201	0.5

Table S1: Structural data, density and band gap of the three proposed layered structures of SiS_2 at 10 GPa calculated with PBE functional.

Elastic properties

Elastic constants $C_{\alpha\beta}$ were calculated using VASP by calculating stress for distorted supercells (no ionic relaxation) and for undistorted supercells (with ionic shifts). For each phase, we used 21 different values of strain - from 0.005 to 0.025 in 0.001 intervals. From these, only results that were quite similar (formed plateaus on $C_{\alpha\beta}$ vs. strain dependences) were used to obtain final average - this was from strain values 0.015 to 0.02 for $P\bar{3}m1$ phase, between 0.011-0.021 for the case of $P6_3mc$ and for 0.013-0.016 interval for $R\bar{3}m$.

Laue group association with crystallographic space group determines the number of independent elastic constants (and relations between the others) of a crystalline material. For the most stable $P\bar{3}m1$ phase, for example, the point group associated with it is $\bar{3}m$ and the Laue group is $\bar{3}m$, denoted as R I. This phase has six independent second-order elastic constants - C_{11}, C_{33}, C_{12}, C_{13}, C_{14} and C_{44} (listed in the fourth column), while other values of $C_{\alpha\beta}$ are given by simple algebraic relations (second column). Those constants not listed in the table equal zero from symmetry constraints.

Mechanical stability of crystals can be predicted according to Born stability criteria coming from the requirement of positive definiteness of $C_{\alpha\beta}$ matrix of elastic constants. Applying this requirement for each of the Laue groups associated with each space group, one obtains specific inequalities as the stability conditions for every crystal according to its point group. For our investigated phases, these are listed in the forth column.

phase (space group)	point group associated with space group and $C_{\alpha\beta}$ symmetry relations	Laue group	independent elastic constants $C_{\alpha\beta}$ [GPa]	stability conditions
$P\bar{3}m1$	$\bar{3}m$	R I ($\bar{3}m$)	6	
	$C_{22} = C_{11}$<br>$C_{23} = C_{13}$<br>$C_{24} = -C_{14}$<br>$C_{55} = C_{44}$; $C_{66} = \frac{1}{2}(C_{11} - C_{12})$<br>$C_{56} = C_{14}$		$C_{11} = 192.8$; $C_{33} = 25.4$<br>$C_{12} = 42.0$; $C_{13} = 5.6$<br>$C_{14} = 0.2$<br>$C_{44} = 7.7$	$C_{11} > \|C_{12}\|$<br>$2C_{13}^2 < C_{33}(C_{11} + C_{12})$<br>$2C_{14}^2 < C_{44}(C_{11} - C_{12})$<br>$C_{44} > 0$
$P6_3mc$	$6mm$	H I ($6/mmm$)	5	
	$C_{22} = C_{11}$<br>$C_{23} = C_{13}$<br>$C_{55} = C_{44}$; $C_{66} = \frac{1}{2}(C_{11} - C_{12})$		$C_{11} = 191.5$; $C_{33} = 25.3$<br>$C_{12} = 41.2$; $C_{13} = 5.2$<br>$C_{44} = 6.7$	$C_{11} > \|C_{12}\|$<br>$2C_{13}^2 < C_{33}(C_{11} + C_{12})$<br>$C_{44} > 0$<br>$C_{66} > 0$
$R\bar{3}m$	$\bar{3}m$	R I ($\bar{3}m$)	6	
			$C_{11} = 190.9$; $C_{33} = 22.4$<br>$C_{12} = 40.0$; $C_{13} = 5.6$<br>$C_{14} = 0.1$<br>$C_{44} = 4.1$	

Table S2: Elastic constants $C_{\alpha\beta}$ of the layered forms at zero pressure and temperature, symmetry relations and Born stability conditions. Note that the Laue groups of $P\bar{3}m1$ and $R\bar{3}m$ space groups are the same.
